# Hardships of Farmers’ Wives

**Published:** 1882-05

**Authors:** 


### HARDSHIPS OF FARMERS' WIVES.

A sad record is it, and short! but its details would fill whole shelves of a library, more intensely interesting than any tale fancy ever told, as found in an official report for 1862, made to the Legislature of an agricultural State, that of six hundred and seven patients in an insane asylum, thirty-nine were farmers' wives, sixteen farmers' daughters; no other class of wives or daughters was half as numerous! and this in spite of all that has been said and sung of the dairy-maid, so ruddy of cheek, where the roses and the lily vie, so lithe of limb, whose breath, as pure as the air of the morning, whose laugh, as merry as the voices of the birds in the wood, and whose step as springy and elastic as the new-made bow; all these bright fancies vanish like mists of the morning before a summer's sun, in face of the hard, dry, statistical line written above; and there comes up another vision, not of youth and beauty and innocence and exuberant health, but that of the pale and wan and haggard face, half covered with long black hair, and coal-black eyes peering hotly on you from behind the bars and grates of a dark prison-house! True, happily true is it, that this state of things is "not to the manor born;" is not part and parcel, necessarily, of the farm-house; it is not an inherent calamity; it is only an accidental circumstance, which can be remedied promptly and forever! and it is because of this delightful truth these pages are written.

The remedy for the startling evil which the official statement made in the beginning brings to light is in the husband and mother. Let the farmer feel that his wife is an equal partner on the farm, and as such is entitled to as high consideration as he claims to himself. He should have a jealous care for the "good name of the house." Let him feel that what degrades his wife degrades himself, that whatever weakens her authority weakens his own power of success; and that in the great struggle of life they must of necessity rise or fall together. But while he cherishes these views as a business matter, as a practical thing of profit and loss, let him make an effort in another direction, not considering his wife merely as his partner in business, but as the love of his youth, who having, in a perfect abandon of trustingness, thrown herself in his strong arms to guide and protect and sustain through life's long journey, has claims on him for these, stronger than any tie other than that which binds man to Divinity; so strong, indeed, that inspiration has declared it, that in heaven was it made, and by heaven only can it be unloosed; and feeling thus, let him see in the wife of his bosom, though she may be all wrinkled with age, only the fair and loving and the fondly trusting girl as she appeared at the moment of saying "I will" many years ago. Let the mother also busy herself in teaching her daughters what they ought to do, what they have a right to expect in the marriage relation; and above this even, let her inculcate on her sons, day by day, with wisdom and tenderness, charging them lovingly to remember when she is dead and gone, that by all the respect and reverence and affection which they may have for her memory, to treat for her sake the wife of their bosom with all that affection and tenderness and consideration and sympathy which they would have their father show her if she could but be brought back again, or which they themselves would gladly show her if they had the opportunity.

Many a farmer's wife is literally worked to death in an inadvertent manner; from want of reflection or consideration on the part of her husband. None can understand better than he, in plowing or sowing, or harvest-time, that if a horse gets sick, or runs away, or is stolen, another must be procured that very day, or the work will inevitably go behind-hand. He does not carry the same practical sense into the kitchen when the hired help leaves without warning, or becomes disabled; although he knows as well as any man can know, that "the hands" will expect their meals with the same regularity, with the same promptness, and with the same proper mode of preparation; but instead of procuring other "help" on the instant, he allows himself to be persuaded, if the "help" is sick, she will get well in a day or two, or week at farthest, and that it is hardly worth while to get another for so short a time; if the "help" has taken "French leave," his mind fixes on the fact that it is a very busy time, and neither he nor a single hand can be spared; or that, in the course of a week, some one will have to go to town for some other purpose, and both these matters can be attended to at the same time; meanwhile, the wife is expected not only to attend to her ordinary duties as usual, but somehow or other to spare time to do all that the cook or washerwoman was accustomed to; that is, to do the full work of two persons, each one of whom had already quite as much labor to perform as she could possibly attend to. The wife attempts it; by herculean efforts all goes on well; the farmer perceives no jar, no hitch, in the working of the machinery; and because no complaint is uttered, thinks that every thing is going on without an effort; meanwhile time passes—and infinite shame on some of them!—they begin to calculate how much has been saved from servants' wages, and how much less food has been eaten; and because still no complaint is made, the resolution quietly forms in the mind to do nothing until she does complain; but, before that takes place, she falls a victim to her over-exertions, in having laid the foundation for weeks and months of illness, if not of a premature decline and death. Sincerely it is believed that these statements ought to be written in large letters above the mantles of half the farmers of the country; and if over the other half also, it would not be labor lost in favor of many a heroic and uncomplaining, but outraged farmer's wife and daughter.

The plain fact is, no farmer who is even half a man, will allow himself to have any help on the farm until he has first supplied a "help" for his wife in the kitchen; for when any man sets out to make a living by farming, there is always quite enough for two women to do,—even if there are no children, nor any prospect of any. No one woman can possibly do all the work of a farm which belongs to her department, even if the only "hand" is her husband; for there is cooking and washing, and sewing and mending, and beds to make; rooms to sweep and floors to wash, and cows to be milked, and butter to be made, and the small gardening to be attended to, and provision to be made for the company that will occasionally call in, and that ought to call in, to break the tedium of the livelong day, the husband in the fields, and not a living soul to exchange a human word to! Whatever young farmer thinks that one woman could or ought to attend to all these matters, even on a one-hand farm, is utterly destitute of all chivalry, and disgraces his own manhood, in that it looks strongly to the fact that he considers his wife more a kind of beast of burden than a rational, social, and intellectual being.

The generous-hearted and manly farmer will steadily bear in mind the great practical fact, that only half his duty has been performed toward the wife of his bosom when he has removed the physical hardships of her lot as far as his means will allow, for these bear chiefly on her physical nature in the direction of shortening her life by but a few years only; the greater evil of a living death behind the bars of a mad-house is the result.

